# DXA-measured truncal adiposity in adolescence but not in childhood longitudinally predicts worsening cardiac outcomes

**DOI:** 10.1093/ejendo/lvag044

**Published:** 2026-04-14

**Authors:** Andrew O Agbaje

**Affiliations:** Institute of Public Health and Clinical Nutrition, School of Medicine, Faculty of Health Sciences, University of Eastern Finland, Kuopio 70211, Finland; Department of Public Health and Sports Sciences, Faculty of Health and Life Sciences, Children's Health and Exercise Research Centre, University of Exeter, Exeter EX1 2LU, United Kingdom

**Keywords:** obesity, pediatrics, preventive cardiology, hypertension, health promotion

## Abstract

**Background:**

Longitudinal evidence on the dual-energy X-ray absorptiometry measures of body composition from childhood with changes in cardiac structure and function in youth is scarce.

**Objectives:**

This study examined whether the increase in total and trunk fat mass during growth from childhood is associated with progressive cardiac remodeling and whether increased systolic blood pressure and inflammation explained the associations.

**Methods:**

From the Avon Longitudinal Study of Parents and Children (ALSPAC), UK birth cohort, 1803 children aged 9 years who had repeated dual-energy X-ray absorptiometry-measured fat mass at ages, 9, 11, 15, and 17 and 24 years clinic visits were included. Echocardiography at 17 and 24 years assessed left ventricular mass indexed for height^2.7^ (LVM).

**Results:**

Each unit increase of total fat mass from age 9-24 years (*β* = −0.48 g/m^2.7^ [95% CI, −0.50-−0.48], *P* < .001) and trunk fat mass (−0.78 g/m^2.7^ [−0.83 to −0.74], *P* < .001) were independently associated with progressively decreased LVM over 7 years. However, each unit increase of total fat mass from age 17-24 years (*β* = 0.12 g/m^2.7^ [95% CI, 0.09-0.15], *P* < .001) and trunk fat mass (0.23 g/m^2.7^ [0.18-0.28], *P* < .001) were independently associated with increased LVM. Each unit increase of lean mass from age 9-24 years (*β* = 0.31 g/m^2.7^ [95% CI, 0.29-0.33], *P* < .001) and body mass index (0.24 g/m^2.7^ [0.20-0.27], *P* < .001) were independently associated with increased LVM. Increased systolic blood pressure and high-sensitivity C-reactive protein partly mediated (10.6% and 7.4% mediation, respectively), the association between increased fat mass and increased cardiac mass during growth from adolescence to young adulthood.

**Conclusions:**

Increased total and trunk fat mass from adolescence but not from childhood predicted progressive cardiac structural and functional pathologies by young adulthood.

## Graphical Abstract

**Graphical Abstract:**
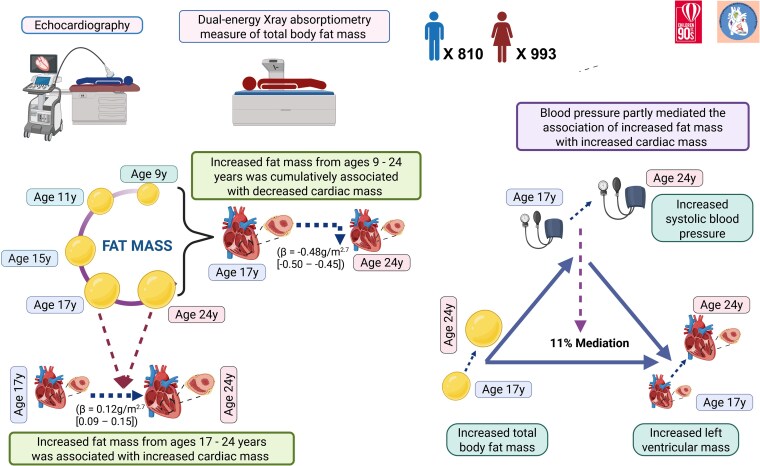

Significance This study assessed for the first time the long-term effects of directly measured total body fat mass and trunk fat mass during growth from childhood through young adulthood, with changes in cardiac structure and function. The cardiac effect of a 15-year-long increase in lean mass was also examined. The study revealed that accumulated total and trunk fat mass in late adolescence, but not in childhood, was adversely associated with cardiac outcomes in early adulthood. Trunk fat mass had a 2-fold adverse effect on the heart compared with total fat mass during growth from adolescence to young adulthood. Increased fat mass may increase systolic blood pressure and inflammation, which could adversely affect the young heart. Body mass index increase was positively associated with cardiac changes, but this was largely explained by increased lean mass from childhood. The increased lean mass association with increased cardiac mass may be considered physiologic and cardio-protective. Lowering total fat mass and trunk fat mass to childhood levels may improve heart health.

## Introduction

While the global prevalence of obesity in the young population is increasing, obesity in childhood and adolescence remains misdiagnosed due to inadequate measures, such as body mass index (BMI).^1-7^ The dual-energy X-ray absorptiometry is considered a highly reliable measure of body composition and is able to distinguish between fat mass and lean mass, thereby overcoming several limitations of the BMI in relation to health outcomes in the young population.^2-4,7-9^ The scarcity of long-term repeated dual-energy X-ray absorptiometry measures of body composition has significantly hampered evidence on the true effect of body composition on cardiovascular physiology and pathologies.^5-7,10-15^

Large-scale longitudinal studies in children and adolescents have reported that lean mass but not fat mass drives arterial wall remodeling.^4,16^ In 2018, it was reported among 32 lean and 15 adolescents with obesity from Germany, followed up for 3 years, that weight reduction (BMI) was associated with improved obesity-induced structural and functional cardiac alterations.^17^ A meta-analysis of 70 cross-sectional studies (9983 children and adolescents) concluded in 2021 that higher BMI was associated with worse cardiac function.^18^ In 2023, a cross-sectional study among 82 adolescents from the UK reported that the associations between BMI and early cardiac remodeling were mediated predominantly by nonadipose tissue.^19^ A cross-sectional study of 2836 children from the Netherlands, aged 10 years, with dual-energy X-ray absorptiometry measures of body composition and cardiac magnetic resonance imaging scans concluded that higher childhood BMI was associated with a larger right and left ventricular size, which was largely influenced by higher lean mass.^20^ The study further noted that lean mass may be a stronger determinant of heart growth than fat mass, with fat mass influencing cardiac structures at older ages.^20^ Another cross-sectional study of 201 US children and adolescents aged 6 to 17 years with dual-energy X-ray absorptiometry measures of body composition in relation to cardiac mass concluded that lean body mass alone explained 75% of the variance of left ventricular mass (LVM), with fat mass and systolic blood pressure explaining only 1.5% and 0.5% of the variance, respectively.^21^ These studies highlighted the scarcity of longitudinal studies that would clarify the relationship between body composition and pediatric cardiovascular systems.^18,19^

Recently, a study among 60-64-year-old adults concluded that increased BMI in adulthood was associated with adverse cardiac structure and function, while a previous Mendelian randomization study reported that BMI might be causally related to LVM.^11,22^ Among adults, higher LVM is a strong predictor of cardiovascular mortality, and regressive changes in LVM have been associated with low rates of clinical events.^23,24^ In the pediatric population, left ventricular hypertrophy serves as a marker of early cardiac damage due to the rarity of clinical events.^25-29^ We and others have observed a late adolescence increase in metabolic parameters following a significant natural decrease postpuberty.^30-33^ Moreover, we have identified that vascular alterations are significantly deleterious in late adolescence compared with midadolescence and childhood.^32,33^ Nonetheless, studies examining whether childhood and adolescent lean mass drives changes in cardiac indices and when fat mass exerts a deleterious effect on cardiac structure and function in youth are nonexistent.

The mechanism through which fat mass influences cardiac structure and function during growth from adolescence through young adulthood is not fully known.^5,6,34,35^ Basic studies have reported that obesity-induced inflammation (adipokine hypothesis) and dyslipidemic state may increase systemic oxidative stress and proinflammatory markers, resulting in cardiac fibrosis and heart failure.^5,6,14,36,37^ The present study examined (1) the separate longitudinal associations of total fat mass, trunk fat mass, lean mass, and BMI from ages 9-24 years and age 17-24 years with changes in cardiac structure and function from ages 17-24 and at age 24 years, (2) the mediating roles of lipids, insulin resistance, high-sensitivity C-reactive protein (CRP), and systolic blood pressure in the associations of fat mass with cardiac indices using data from the Avon Longitudinal Study of Parents and Children birth cohort, England, UK. We hypothesize that increased adiposity and lean mass during childhood and postpubertal adolescence will similarly influence cardiac structural and functional indices.

## Methods

Details of the Avon Longitudinal Study of Parents and Children birth cohort have been published earlier.^38-41^ The birth cohort investigates factors that influence childhood development and growth. Altogether, 14 541 pregnancies from women residing in Avon, southwestern England, UK, who had a total of 14 676 fetuses, were enrolled between April, 1, 1991, and December, 31, 1992. Of these 13 988 children were alive at 1 year of age. From age 7, an attempt was made to bolster the initial sample with eligible cases who had failed to join the study originally. The phases of enrollment are described in more detail in the cohort profile paper and its update.^38,40^ The total sample size from the age of 7 is therefore 15 447 pregnancies, resulting in 15 658 fetuses, of which 14 901 children were alive at 1 year of age. Annual face-to-face clinics commenced from the age of 7 and are ongoing. Altogether 2079 participants had cardiac structural measures at the age 17-year clinic visit and 1957 participants had cardiac measures at 24 years. For the current analysis, 1803 participants who had complete cardiac at age 17 years and repeated dual-energy X-ray absorptiometry measures at ages 9, 11, 15, 17, and 24 years were included in the study. Ethical approval for the study was obtained from the Avon Longitudinal Study of Parents and Children Ethics and Law Committee and the Local Research Ethics Committees. Informed consent for the use of data collected via questionnaires and clinics was obtained from participants following the recommendations of the Avon Longitudinal Study of Parents and Children Ethics and Law Committee at the time. Study data at 24 years were collected and managed using REDCap electronic data capture tools.^42^ All research complied with the Declaration of Helsinki.

### Anthropometric, cardiometabolic, and lifestyle factors measures

Body composition (total fat mass, trunk fat mass, and lean mass) was assessed using a dual-energy X-ray absorptiometry scanner (GE Medical Systems, Madison, Wisconsin) at age 9, 11, 15, 17, and 24-year clinic visits. Repeated dual-energy X-ray absorptiometry measurements for 122 children were performed on the same day, and the repeatability co-efficient (twice the standard deviation of the difference between measurement occasions) for body fat mass was 0.5 kg.^3,8,9^ Dual-energy X-ray absorptiometry measurement of body composition is highly correlated with magnetic resonance imaging measure in children (97% correlation).^43^ Height was measured to the nearest 0.1 cm with Harpenden wall-mounted stadiometer (Holtain Ltd, Crosswell, Crymych, UK), weight to the nearest 0.1 kg was measured using (Tanita TBF-401 Model A, Tanita Corp., Tokyo, Japan electronic scale), BMI was computed as weight in kilograms per height in meters squared.^3,8,9^ Overweight/obesity was categorized as BMI >24.99 kg/m^2^ in late adolescence and young adulthood. Heart rate and systolic and diastolic blood pressure were measured at ages 17 and 24 years using an Omron 705-IT as previously detailed. Fasting blood samples at ages 17 and 24 years were collected, spun, and frozen at −80 °C and later assayed for glucose and insulin, as detailed previously. Fasting insulin was measured using an ultrasensitive, automated microparticle enzyme immunoassay (Mercodia), which does not cross-react with proinsulin, and the sensitivity of the immunoassay was 0.07 mU/L. The homeostatic model assessment of insulin resistance was calculated from (fasting insulin × fasting glucose/22.5).^44^ Fasting plasma triglyceride, high-density lipoprotein cholesterol, low-density lipoprotein cholesterol, and high-sensitivity CRP were assessed at both 17 and 24-year clinic visits in line with standard protocols. Questionnaires to assess smoking behavior were administered at the 17-year and 24-year clinic visits.^45^ At the 17-year clinic visit, participants were briefly asked about their personal and family (mother, father, and siblings) medical history, such as a history of hypertension, diabetes, high cholesterol, and vascular disease.^46^ The participant's mother's socioeconomic status was grouped according to the 1991 British Office of Population and Census Statistics classification.^4^ Sedentary time, light physical activity, and moderate-to-vigorous physical activity in adolescence and young adulthood were assessed with ActiGraph accelerometer worn for 4-7 days.

### Cardiac structure and function measures

At 17 years, echocardiography was performed according to American Society of Echocardiography guidelines^47,48^ by 1 of 2 experienced echocardiographers using an HDI 5000 ultrasound machine (Phillips Healthcare, Amsterdam, The Netherlands) equipped with a P4-2 Phased Array ultrasound transducer. At 24 years, echocardiography was performed by 2 experienced echocardiographers using a Philips EPIQ 7G Ultrasound System equipped with a ×5-1 transducer. Philips Q-station was used for the M-mode, 2D, and Doppler echo analyses, while TomTec software was used for the 3D echo analyses. Measures of cardiac structure were LVM allometrically scaled to height^2.7^ and relative wall thickness (RWT) computed from septal wall thickness, posterior wall thickness, and left ventricular diastolic diameter.^49^ Measures of cardiac function were left ventricular diastolic function (LVDF) E/A wave ratio and left ventricular filling pressure (LVFP) E/e′ wave ratio. Pulsed Doppler examination of transmitral flow was recorded from the apical 4-chamber view. For LV measurements, the sample volume was positioned between the mitral annulus and the tips of the mitral leaflets, with the position adjusted to maintain the sample volume at an angle as near parallel to transmitral flow as possible with the participant in passive end-expiration.^47^ The peak flow velocity of the early (E) and atrial (A) waves were measured from the 3 consecutive cardiac cycles displaying the highest measurable velocity profiles.^47^ Similar measurement (e′) was conducted at the tricuspid valve. Tissue Doppler echocardiography was performed in the 4-chamber view on the lateral, inferior, and septal LV walls to obtain myocardial wall velocities. Data were acquired with the beam parallel to the wall of interest and with optimal settings to ensure no over-gain of the low-velocity signals. A 5 mm sample volume was placed at the level of the mitral valve annulus, and a loop of 8-10 cardiac cycles was recorded. The reproducibility of echocardiographic examinations was assessed by recalling 30 participants and repeating their measurements. The intraclass correlation of repeated measurements ranged from 0.75 to 0.93 (intraobserver) and 0.78 to 0.93 (interobserver).^49,50^

### Statistical analysis

Participant's descriptive characteristics were summarized as means and standard deviation, medians and interquartile ranges, or frequencies and percentages. We explored sex differences using independent t-tests, Mann-Whitney U tests, or Chi-square tests for normally distributed, skewed or dichotomous variables, respectively. We assessed the normality of variables and logarithmically or reciprocally transformed skewed variables prior to further analyses.

The separate associations of the 15-year increase in total fat mass, trunk fat mass, lean mass, and BMI repeatedly measured at ages 9, 11, 15, 17, and 24 years with each of the progressive changes in LVM, RWT, LVDF, and LVFP from ages 17 to 24 years were examined using generalized linear mixed-effect models for repeated measures. Model 1 was unadjusted. Model 2 was adjusted for sex and other time-varying covariates measured at both baseline and follow-up such as age, high-sensitivity CRP, heart rate, systolic blood pressure, smoking status, family history of hypertension/diabetes/high cholesterol/vascular disease, socioeconomic status, sedentary time, light physical activity, moderate-to-vigorous physical activity, low-density lipoprotein cholesterol, high-density lipoprotein cholesterol, or triglyceride, insulin resistance, and fat mass or lean mass depending on the predictor. Additional generalized linear mixed-effects model analyses were separately conducted for body composition measured between ages 9-17 years or 17-24 years in relation to cardiac functional and structural outcomes measured from ages 17 to 24 years.

Mediating path analyses using structural equation models separately examined the mediating role of each of cumulative low-density lipoprotein cholesterol, triglyceride, high-sensitivity CRP, insulin resistance, and systolic blood pressure on the longitudinal associations of each of cumulative total fat mass from age 17-24 years with the progression in LVM. The mediation analysis was conducted in line with the Guideline for Reporting Mediation Analyses of Randomized Trials and Observational Studies (AGReMA).^51^ The examined mediation mechanism between total fat mass and LVM is partly based on previous studies in which worsening cardiometabolic profiles have been associated with cardiac diseases.^5,6^ Analyses were adjusted for sex, family history of hypertension/diabetes/high cholesterol/vascular disease, socioeconomic status, and time-varying covariates measured at both baseline and follow-up such as age, heart rate, smoking status, sedentary time, light physical activity, moderate-to-vigorous physical activity, high-density lipoprotein cholesterol, low-density lipoprotein cholesterol, triglyceride, insulin resistance, lean mass, high-sensitivity CRP, depending on the predictor or mediator.

The path models had 3 equations per regression analysis: the longitudinal associations of cumulative total fat mass with cumulative LVM (Equation 1); the longitudinal associations of cumulative low-density lipoprotein cholesterol, triglyceride, high sensitivity CRP, systolic blood pressure, and insulin resistance with LVM (Equation 2); and the longitudinal associations of cumulative total fat mass with cumulative LVM (Equation 3, total effect), and Equation 3′ (direct effect) accounting for the mediating effect of low-density lipoprotein cholesterol, triglyceride, high sensitivity CRP, systolic blood pressure, and insulin resistance. The proportion of mediating or suppressing roles was estimated as the ratio of the difference between Equation 3 and Equation 3′ or the multiplication of Equations 1 and 2 divided by Equation 3 and expressed in percentage. A mediating or indirect role is confirmed when there are statistically significant associations between (a) the predictor and mediator, (b) the predictor and outcome, (c) the mediator and outcome, and (d) the longitudinal association between the predictor and outcome variable attenuates upon inclusion of the mediator.^52^ A statistically significant mediation of <1% is considered minimal, and ≥1% as partial. Path analyses were conducted with 1000 bootstrapped samples.^53,54^

Differences and associations with a 2-sided *P*-value <.05 were considered statistically significant with conclusions based on effect estimates and their confidence intervals or standard errors. Analyses involving 20% of a sample of 10 000 Avon Longitudinal Study of Parents and Children birth cohort participants at 0.8 statistical power, 0.05 alpha, and 2-sided *P*-value would show a minimum detectable effect size of .062 standard deviations if they had relevant exposure for a normally distributed quantitative variable.^55^ Missing data were handled with 20 cycles of multiple imputations as it has >98% efficiency in simulating real data in our cohort.^8^ Multiple comparisons were adjusted for using sequential Sidak correction. All statistical analyses were performed using SPSS statistics software, Version 29.0 (IBM Corp, Armonk, NY, USA), and mediation analyses was performed with IBM AMOS version 27.

## Results

### Cohort study characteristics

**Figure 1:**
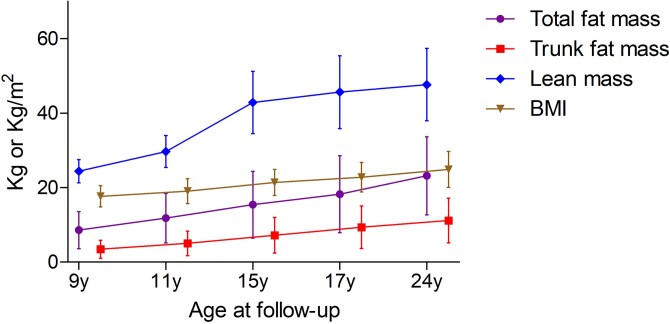
Mean (standard deviation) trajectory of body composition from childhood through young adulthood from the Avon Longitudinal Study of Parents and Children birth cohort, England, UK. BMI, body mass index (kg/m^2^). Total fat mass, trunk fat mass, and lean mass are expressed in kg.

Among 1803 children (mean [SD] age, 9.82 [0.30] years; 993 [55.1%] females), total fat mass, trunk fat mass, lean mass, and BMI increased during growth from childhood through young adulthood (Figure 1, Table 1 and Table S1). The average LVM increase during growth from adolescence to young adulthood is 3 g/m^2.7^. Other characteristics are described in Table 1.

**Table 1:** Descriptive characteristics of cohort participants.

	17 years			24 years		
Variables	Male (*n* = 810)	Female (*n* = 993)	*P*-value	Male	Female	*P-value*
	Mean (SD)	Mean (SD)		Mean (SD)	Mean (SD)	
***Anthropometry***						
Age at clinic visit (years)	17.72 (0.35)	17.76 (0.41)	.116	24.40 (0.56)	24.37 (0.64)	.*330*
Height (m)	1.79 (0.07)	1.66 (0.06)	<.001	1.80 (0.07)	1.66 (0.06)	*<*.*001*
*Weight (kg)	69.6 (12.0)	59.95 (12.67)	<.0001	78.0 (15.6)	63.8 (15.5)	*<*.*0001*
Ethnicity- Caucasian (*n*, %)	581 (97.2)	834 (95.8)	0.204	NA	NA	*NA*
***Body composition***						
*Total body fat mass (kg)	10.72 (9.12)	18.58 (9.68)	<.0001	18.25 (8.85)	21.13 (10.2)	*<*.*0001*
*Trunk fat mass (kg)	5.56 (6.48)	9.54 (5.70)	<.001	8.80 (6.48)	9.86 (7.37)	*<*.*001*
*Lean mass (kg)	54.75 (8.33)	37.89 (4.68)	<.0001	55.9 (10.09)	40.86 (6.40)	*<*.*0001*
*Body mass index (kg/m^2^)	21.39 (3.51)	21.79 (4.07)	.079	23.73 (4.21)	23.05 (4.83)	.*004*
Overweight and obese ≥25 kg/m^2^ (*n*, %)	75 (13.4)	139 (17.1)	.069	210 (32.8)	675 (29.3)	.*150*
***Fasting plasma metabolic indices***						
High-density lipoprotein (mmol/L)	1.21 (0.26)	1.38 (0.31)	<.001	1.42 (0.36)	1.69 (0.42)	*<*.*001*
Low-density lipoprotein (mmol/L)	1.97 (0.58)	2.23 (0.64)	<.001	2.37 (0.74)	2.37 (0.73)	.*940*
*Triglyceride (mmol/L)	0.72 (0.30)	0.74 (0.37)	.198	0.86 (0.46)	0.80 (0.43)	*<*.*001*
Glucose (mmol/L)	5.14 (0.44)	4.90 (0.37)	<.001	5.54 (0.90)	5.23 (0.54)	*<*.*001*
*Insulin (mU/L)	5.74 (3.87)	7.27 (4.51)	<.001	7.08 (4.56)	7.70 (5.34)	*<*.*001*
*Homeostatic model assessment for insulin resistance	1.38 (1.02)	1.67 (1.11)	<.001	1.75 (1.26)	1.86 (1.48)	.*102*
*High-sensitivity C-reactive protein (mg/L)	0.43 (0.58)	0.67 (1.41)	<.001	0.57 (0.96)	0.92 (1.82)	*<*.*001*
***Vascular measures***						
Heart rate (beat/mins)	63 (9)	67 (10)	<.001	63 (10)	68 (10)	*<*.*001*
Systolic blood pressure (mmHg)	119 (9)	110 (8)	<.001	122 (10)	111 (10)	*<*.*001*
Diastolic blood pressure (mmHg)	63 (6)	65 (6)	<.001	66 (7)	66 (7)	.*049*
***Cardiac measures***						
Left ventricular mass indexed for height (g/m^2.7^)	36.85 (7.76)	34.30 (6.77)	<.001	40.82 (8.70)	36.34 (7.92)	*<*.*001*
Relative wall thickness (cm)	0.38 (0.06)	0.36 (0.06)	.273	0.37 (0.06)	0.36 (0.06)	.*003*
Left ventricular diastolic function (E/A)	1.96 (0.38)	1.93 (0.38)	.333	1.96 (0.52)	2.02 (0.60)	.*059*
Left ventricular filling pressure (E/e′)	4.84 (1.20)	4.87 (0.92)	<.001	4.87 (0.99)	5.13 (1.03)	.*664*
***Lifestyle factors***						
Smoked cigarette in the last 30 days (*n*, %)	113 (23)	185 (25.9)	.277	174 (27.6)	243 (25.6)	.*382*
Family history of H-D-C-V (*n*, %)	157 (28.4)	251 (31.5)	.229	NA	NA	*NA*
Sedentary time (min/day)	467 (86)	486 (78)	.003	533 (83)	526 (86)	.*374*
Light physical activity (min/day)	285 (67)	272 (60)	.007	145 (54)	148 (51)	.*451*
Moderate to vigorous physical activity (min/day)	56 (32)	40 (21)	<.001	57 (35)	47 (28)	*<*.*001*
Maternal social economic status (*n*, %)			.181			
*Professional occupation*	34 (11)	19 (4.5)		NA	NA	*NA*
*Managerial and technical*	127 (41.1)	178 (42.5)				
*Skilled nonmanual*	97 (31.4)	152 (36.3)				
*Skilled manual*	<7 (1.3)	7 (1.7)				
*Partly skilled*	41 (13.3)	54 (12.9)				
*Unskilled*	<7 (1.9)	9 (2.1)				

The values are means (standard deviations) and *median (interquartile range) except for lifestyle factors and ethnicity. Differences between sexes were tested using Student's t-test for normally distributed continuous variables, Mann-Whitney U test for skewed continuous variables, Chi-square test for dichotomous variables, and analysis of covariance for multicategory variables. A 2-sided *P*-value <.05 is considered statistically significant. NA, not available/applicable; *P*-value for sex differences separately at baseline and at follow-up. H-D-C-V hypertension/diabetes/high cholesterol/vascular disease.

### Longitudinal effect of body composition from ages 9 to 24 years with changes in cardiac structure and function from age 17 to 24 years

Increased total fat mass and trunk fat mass from ages 9-24 years were associated with progressively decreased LVM and LVDF, and increased RWT and LVFP from ages 17-24 years after full adjustments (Table 2). Increased lean mass from ages 9-24 years was associated with progressively increased LVM and RWT but decreased LVDF and LVFP from ages 17-24 years (Table 2). Increased BMI from ages 9-24 years was associated with progressively increased LVM, LVDF and LVFP but decreased RWT from ages 17-24 years (Table 2).

**Table 2:** Longitudinal associations of the cumulative increase in body composition from ages 9, 11, 15, 17, through 24 years with changes in left ventricular mass and diastolic function from ages 17 through 24 years.

*N* = 1803	LVM (g/m^2.7^)		RWT (cm)		LVDF (E/A)		LVFP (E/e′)	
*Ages 9-24 years*	*β (95% CI)*	*P-value*	*β (95% CI)*	*P-value*	*β (95% CI)*	*P-value*	*β (95% CI)*	*P-value*
**Total body fat mass (kg)**								
*Model 1*	−0.016 (−0.038-0.005)	.142	0.001 (0.001-0.001)	**<**.**001**	−0.005 (−0.006-−0.004)	**<**.**001**	0.004 (0.002-0.006)	**<**.**001**
*Model 2*	−0.475 (−0.501-−0.450)	**<**.**001**	0.001 (0.001-0.001)	**<**.**001**	−0.002 (−0.003-−0.001)	**<**.**001**	0.008 (0.006-0.010)	**<**.**001**
**Trunk fat mass (kg)**								
*Model 1*	0.041 (0.002-0.080)	.**039**	0.002 (0.002-0.002)	**<**.**001**	−0.008 (−0.010-−0.007)	**<**.**001**	0.004 (0.001-0.008)	.**016**
*Model 2*	−0.784 (−0.829-−0.739)	**<**.**001**	0.001 (0.001-0.001)	**<**.**001**	−0.004 (−0.005-−0.003)	**<**.**001**	0.012 (0.008-0.015)	**<**.**001**
**Lean mass (kg)**								
*Model 1*	0.315 (0.298-0.332)	**<**.**001**	0.001 (0.001-0.001)	**<**.**001**	−0.003 (−0.004-−0.003)	**<**.**001**	−0.003 (−0.004-−0.001)	**<**.**001**
*Model 2*	0.311 (0.288-0.333)	**<**.**001**	0.001 (0.000-0.001)	**<**.**001**	−0.004 (−0.005-−0.003)	**<**.**001**	−0.005 (−0.007-−0.004)	**<**.**001**
**Body mass index (kg/m^2^)**								
*Model 1*	0.348 (0.325-0.372)	**<**.**001**	−0.0001 (0.000-0.001)	.061	0.003 (0.002-0.004)	**<**.**001**	0.001 (−0.0001-0.002)	.071
*Model 2*	0.236 (0.202-0.270)	**<**.**001**	−0.001 (−0.001-−0.001)	**<**.**001**	0.003 (0.002-0.004)	**<**.**001**	0.007 (0.005-0.009)	**<**.**001**

Model 1 was unadjusted. Model 2 was adjusted for sex and other time-varying covariates measured at both baseline and follow-up such as age, high-sensitivity C reactive protein, heart rate, systolic blood pressure, insulin, glucose, smoking status, family history of hypertension/diabetes/high cholesterol/vascular disease, socioeconomic status, sedentary time, light physical activity, moderate to vigorous physical activity, low-density lipoprotein cholesterol, high-density lipoprotein cholesterol, triglyceride and fat mass or lean mass, depending on the predictor. When body mass index was the predictor, there were no further adjustments for fat mass or lean mass. Regression coefficients (*β*) were computed from generalized linear mixed-effect model for repeated measures; CI, confidence interval; LVDF, left ventricular diastolic function; LVFP, left ventricular filling pressure; c, left ventricular mass indexed for height^2.7^; RWT, relative wall thickness, A 2-sided *P*-value <.05 is considered statistically significant and is bolded. Multiple testing was corrected with Sidak correction. Multiple imputations (20-cycles) were used to account for missing variables. A 1-unit cumulative increase in exposure is associated with the point estimate increase in the outcome.

### Longitudinal effect of body composition from ages 9 to 24 years with cardiac structure and function at age 24 years

Increased total fat mass and trunk fat mass from ages 9-24 years were associated with lower LVM and LVDF, but higher RWT at age 24 years after full adjustments (Table S2). Increased lean mass from ages 9-24 years was associated with higher LVM and RWT but lower LVDF and LVFP at age 24 years (Table S2). Increased BMI from ages 9-24 years was associated with higher LVM, LVDF, and LVFP but lower RWT at age 24 years (Table S2).

### Longitudinal effect of body composition from ages 17 to 24 years with cardiac structure and function changes from ages 17 to 24 years

Increased total fat mass and trunk fat mass from ages 17-24 years were associated with progressively increased LVM and RWT but decreased LVDF and LVFP from ages 17-24 years after full adjustments (Table 3). Increased lean mass from ages 17-24 years had a statistically significant association with progressively increased LVM from ages 17-24 years (Table 3).

**Table 3:** Longitudinal associations of increased body composition from ages 17-24 years with changes in left ventricular mass and diastolic function from ages 17 through 24 years.

*N* = 1803	LVM (g/m^2.7^)		RWT (cm)		LVDF (E/A)		LVFP (E/e′)	
*Ages 17-24 years*	*β (95% CI)*	*P-value*	*β (95% CI)*	*P-value*	*β (95% CI)*	*P-value*	*β (95% CI)*	*P-value*
**Total body fat mass (kg)**								
*Model 1*	0.230 (0.198-0.261)	**<**.**001**	0.0001 (0.000-0.000)	.830	−0.003 (−0.005-−0.002)	**<**.**001**	−0.004 (−0.008-0.000)	.053
*Model 2*	0.122 (0.090-0.153)	**<**.**001**	0.000 (0.0001-0.001)	.**021**	−0.005 (−0.007-−0.004)	**<**.**001**	−0.012 (−0.016-−0.008)	**<**.**001**
**Trunk fat mass (kg)**								
*Model 1*	0.380 (0.326-0.435)	**<**.**001**	0.001 (0.000-0.001)	.**005**	−0.008 (−0.011-−0.006)	**<**.**001**	−0.011 (−0.018-−0.004)	.**002**
*Model 2*	0.230 (0.178-0.281)	**<**.**001**	0.001 (0.000-0.001)	.**004**	−0.009 (−0.012-−0.006)	**<**.**001**	−0.023 (−0.030-−0.016)	**<**.**001**
**Lean mass (kg)**								
*Model 1*	0.329 (0.310-0.348)	**<**.**001**	0.000 (0.000-0.0001)	.073	0.005 (0.004-0.006)	**<**.**001**	0.002 (−0.001-0.005)	.140
*Model 2*	0.238 (0.216-0.260)	**<**.**001**	0.000 (−0.000-0.000)	.112	0.001 (0.000-0.002)	.142	0.001 (−0.002-0.004)	.419

Model 1 was unadjusted. Model 2 was adjusted for sex and other time-varying covariates measured at both baseline and follow-up such as age, high-sensitivity CRP, heart rate, systolic blood pressure, insulin, glucose, smoking status, family history of hypertension/diabetes/high cholesterol/vascular disease, socioeconomic status, sedentary time, light physical activity, moderate to vigorous physical activity, low-density lipoprotein cholesterol, high-density lipoprotein cholesterol, triglyceride and fat mass or lean mass, depending on the predictor. Regression coefficients (*β*) were computed from generalized linear mixed-effect model for repeated measures; CI, confidence interval; LVDF, left ventricular diastolic function; LVFP, left ventricular filling pressure; LVM, left ventricular mass indexed for height^2.7^; RWT, relative wall thickness, A 2-sided *P*-value <.05 is considered statistically significant and is bolded. Multiple testing was corrected with Sidak correction. Multiple imputations (20-cycles) were used to account for missing variables. A 1-unit increase in exposure is associated with the point estimate increase in the outcome.

### Longitudinal effect of body composition from ages 17 to 24 years with cardiac structure and function at age 24 years

Increased total fat mass from ages 17-24 years was associated with higher LVM and RWT but lower LVDF at age 24 years after full adjustments (Table S3). Increased trunk fat mass from ages 17-24 years was associated with higher LVM and RWT but lower LVDF and LVFP at age 24 years (Table S3). Increased lean mass from ages 17-24 years had statistically significant associations with higher LVM and RWT at age 24 years (Table S3).

### Longitudinal effect of body composition from ages 9 to 17 years with cardiac structure and function changes from ages 17 to 24 years

Increased total fat mass from ages 9-17 years was associated with progressively decreased LVM, LVDF and LVFP but increased RWT from ages 17-24 years after full adjustments (Table S4). Increased lean mass from ages 9-17 years had a statistically significant association with progressively increased LVM and RWT from ages 17-24 years but decreased LVDF and LVFP (Table S4).

### Mediation effect of insulin resistance, lipids, inflammation, and blood pressure, on the association of total fat mass with cardiac structure during growth from age 17 to 24 years

**Figure 2:**
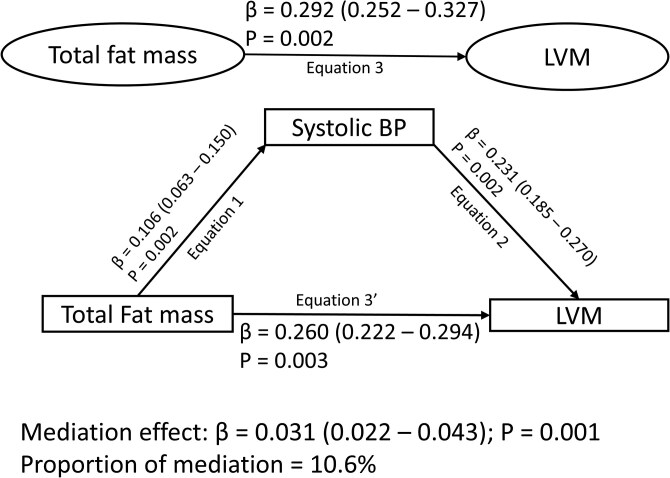
Mediating role of increased systolic blood pressure in the longitudinal relationships of total fat mass with LVM during growth from ages 17 through 24 years. Mediation structural equation model estimating natural direct and indirect effects was adjusted for sex, family history of hypertension/diabetes/high cholesterol/vascular disease, and socioeconomic status, in addition to time-varying covariates such as age, high-sensitivity CRP, heart rate, insulin resistance, smoking status, sedentary time, light physical activity, moderate-to-vigorous physical activity, high-density lipoprotein cholesterol, low-density lipoprotein cholesterol, triglyceride, and lean mass. *β* is standardized regression co-efficient. Two-sided *P*-value < .05 were considered statistically significant; BP, blood pressure; LVM, left ventricular mass indexed for height^2.7^.

Cumulative low-density lipoprotein cholesterol, triglyceride, systolic blood pressure, and high-sensitivity CRP partly and independently mediated the associations of increased total fat mass with increased LVM (2.4%-10.6% mediation effect), but insulin resistance had no statistically significant mediating effect (Table 4 and Figure 2).

**Table 4:** Mediating or suppressing role of cumulative blood pressure, and fasting lipids, inflammation, insulin resistance on the longitudinal associations of increased fat mass and lean mass and left ventricular mass progression from ages 17 through 24 years.

	Cumulative left ventricular mass from ages 17-24 years						
	Total effect		Direct effect		Indirect effect		Mediation or Suppression (%)
*Mediators*	*β (95% CI)*	*P-value*	*β (95% CI)*	*P-value*	*β (95% CI)*	*P-value*	
**Cumulative fat mass 17-24 years**							
Low-density lipoprotein cholesterol	0.266 (0.227-0.300)	.003	0.245 (0.205-0.283)	.002	0.021 (0.013-0.031)	.**001**	**7.9 mediation**
Triglyceride	0.249 (0.212-0.284)	.002	0.244 (0.206-0.279)	.002	0.006 (0.001-0.012)	.**011**	**2.4 mediation**
Systolic blood pressure	0.292 (0.252-0.327)	.002	0.260 (0.222-0.294)	.003	0.031 (0.022-0.043)	.**001**	**10.6 mediation**
High-sensitivity CRP	0.271 (0.235-0.305)	.002	0.251 (0.209-0.289)	.002	0.020 (0.007-0.034)	.**006**	**7.4 mediation**
Insulin resistance	0.273 (0.237-0.307)	.003	0.257 (0.211-0.295)	.003	0.016 (−0.001-0.033)	.054	5.9

Mediation structural equation model was adjusted for sex, family history of hypertension/diabetes/high cholesterol/vascular disease, socioeconomic status, and time-varying covariates measured at both baseline and follow-up such as age, heart rate, smoking status, sedentary time, light physical activity, moderate-to-vigorous physical activity; lean mass, insulin resistance, high sensitivity CRP, high-density lipoprotein cholesterol, and triglyceride depending on the mediator. *β* is standardized regression co-efficient. *P*-value of indirect effect <.05 was considered statistically significant are bolded. When the magnitude of the longitudinal association between the predictor and outcome is decreased, mediation is confirmed. CRP, C-reactive protein.

## Discussion

In a large population of children, prospectively increased total fat mass and trunk fat mass during adolescence through young adulthood, but not in childhood, were associated with adverse changes in cardiac indices. Increased systolic blood pressure, high sensitivity CRP, and low-density lipoprotein cholesterol partly mediated the relationship of increased total fat mass with progressive cardiac structural changes. These findings may help clarify the transition between physiologic and pathologic LV remodeling induced by fat mass, thereby providing potential clinical and public health implications on timely intervention.

### Effect of body composition on changes in cardiac structure and function

Although childhood obesity assessed with BMI has been associated with a 3-fold increased risk of fatal and nonfatal events by midforties, adult risk factor was a strong predictor of adult events, whereas childhood risk factor was seemingly attenuated.^56^ This study suggests that adolescence transitory phase might be integral to the development of cardiovascular disease.^56^ A Mendelian randomization study suggests that adolescent BMI might be causally related to LVM, while increased LVM in adults predicts higher risks of left ventricular hypertrophy and cardiovascular morbidity and mortality.^11,23,24,37,57^ BMI fails to distinguish between lean mass and fat mass; thus, the relationship between BMI-assessed obesity and LVM remains controversial, especially in childhood.^2,5,6^ Large-scale longitudinal studies have reported that lean mass from childhood but not fat mass drives vascular remodeling.^4,16^ Importantly, increased total fat mass and trunk fat mass during the 15-year growth from childhood to young adulthood were associated with a 16%-26% decrease in LVM, suggestive of a cardio-protective effect of fat mass from age 9 through 24 years. However, during the 7-year growth from adolescence (age 17 years) through young adulthood (age 24 years), a 1 kg increase in trunk fat mass and total fat mass were separately associated with increased LVM, ∼0.23 g/m^2.7^ and ∼0.12 g/m^2^, respectively. On average, LVM increased by ∼3 g/m^2.7^ within the 7-year repeat echocardiography. Thus, total fat mass and trunk fat mass independently contributed 4% and 8% to LVM increase, respectively. Adolescence has been identified as the critical time to interrupt the vicious pathological cycle of fat mass and insulin resistance and potentially reverse subclinical atherosclerotic changes induced by dyslipidemia.^9,32^ Childhood BMI is largely driven by lean mass, and findings are misinterpreted as the adverse effect of fat mass on cardiovascular indices, but BMI assessment of obesity in the present study seems to mask the physiologic cardio-protection of low-fat mass in children.

Both total fat mass and trunk fat mass from adolescence were separately associated with increased RWT and LVM from ages 17 through 24 years as well as higher RWT and LVM at age 24 years. Evidence of increased RWT suggests concentric hypertrophy if LVM is increased and concentric remodeling if LVM is normal.^35^ Concentric LVM hypertrophy is a risk factor for cardiac malformation and pathologies.^35^ The current findings on the effect of total fat mass and trunk fat mass on LVM may be clinically significant since a 25.3 unit decrease in LVM was associated with a 22% lower risk of cardiovascular events.^24^ The average total fat mass at age 9 years was 8.6 kg which doubled to 18.2 kg by age 17 years. Similarly, the average trunk fat mass at age 9 years was 3.4 kg which tripled to 9.3 kg by age 17 years. The present study suggests that maintaining adiposity levels around childhood values may significantly contribute to a healthy heart.^28^ In the present study, it was observed that the potential pathway through which fat mass influences cardiac structure included increased systolic blood pressure (11% mediation), inflammation (7% mediation), and low-density lipoprotein cholesterol (8% mediation). Increased systolic blood pressure and lipids in adolescence have been independently associated with premature cardiac damage.^26,58^ In animal studies^36,37^ excessive lipid alters membrane lipid bilayer, intracellular calcium ion regulation, and isoform expression patterns of myosin heavy chain, which could increase myocardial susceptibility to exogenous damage.^37^ In a recent study, increased fat mass significantly mediated the direct effect of insulin resistance on increased cardiac mass in youth, while hyperglycemia was longitudinally associated with premature cardiac damage.^59^

Among adults, left ventricular diastolic dysfunction and high LVFP predict heart failure with preserved and reduced ejection fraction, acute myocardial infarction, and cardiovascular and all-cause mortality.^34,47,48^ Increased total fat mass and trunk fat mass from childhood were associated with decreased LVDF and increased LVFP, while increased total fat mass and trunk fat mass from adolescence were associated with decreased LVDF and LVFP. These findings suggest a fat mass-induced transition in LVFP during growth from childhood through adolescence. In adults, obesity impairs myocardial relaxation and increases the myocyte lengthening load, resulting in reduced and delayed longitudinal motion and e′ velocity.^34^

The relationship between adiposity and structural and functional cardiac changes is time-dependent. This large-scale longitudinal study, the first to analyze the long-term relationship between objectively assessed body composition and cardiac structure in a pediatric/young adult population, suggests that postpubertal adolescence is a critical time point to primarily prevent cardiovascular disease risk. The prevention of obesity and excess adiposity in childhood may be considered primordial, but if missed, adolescence might be the golden opportunity to avert potential pathologic alterations of cardiac indices. This study fills knowledge gaps identified in the recent European Society of Cardiology Clinical Consensus Statement on Obesity and Cardiovascular diseases as well as a similar adult study.^6,22^ Accumulating sedentary time of >6 hours/day during growth from childhood to young adulthood is an independent driver of excessive total body and truncal fat as well as worsening cardiac structural indices.^8,28^ Lifestyle modifications in childhood and adolescence, such as engaging in light exercise for at least 3 hours/day and increasing muscle mass with vigorous exercise, may reverse excessive adiposity, elevated systolic blood pressure, and cardiac alterations.^8,28,60,61^ Thus, policymakers, public experts, parents, and clinicians need to pay more attention to adolescents’ cardiometabolic health, which may potentially be the onset of a cardiovascular disease trajectory that may lead to adverse cardiovascular health consequences in later life.

### Strength and limitations

In a large cohort, extensively phenotype with repeated measures of variables during childhood through young adulthood, the independent effect of fat mass on cardiac indices was delineated from lean mass. An extensive array of confounders was accounted for, but the existence of unmeasured confounders may not be excluded. The study population represents a highly selected cohort of individuals born in the early 1990s, and therefore may not be fully generalizable to current populations, given the substantial changes over time in maternal age, maternal cardiovascular risk profile, and environmental exposures, including lifestyle, diet, physical activity, air pollution, and socioeconomic factors. The participants were mostly Caucasian; thus, findings may not be generalizable to minority ethnic groups. Lastly, observational studies alone may be insufficient to prove causation, necessitating further experimental studies.

## Conclusion

Lean mass from childhood may be a strong determinant of physiologic cardiac structural remodeling while increased total and trunk fat mass in adolescence, but not in childhood, may predict pathological changes in cardiac indices by young adulthood. Trunk fat mass was 2-fold more deleterious to cardiac structure compared with total fat mass. Lowering total fat mass and trunk fat mass to childhood levels may improve heart health. BMI assessment of obesity in childhood is largely driven by lean mass and thus masks the physiologic cardio-protection of relatively low-fat mass in children. Increased systolic blood pressure, lipids, and inflammation are potential pathways through which increased fat mass may contribute to adverse cardiac changes. Adolescence may be a critical time to interrupt and reverse the cardiovascular consequences of excess fat.

## Supplementary Material

lvag044_Supplementary_Data

## Data Availability

The informed consent obtained from ALSPAC participants does not allow the data to be made freely available through any third-party maintained public repository. However, data used for this submission can be made available on request to the ALSPAC Executive. The ALSPAC data management plan describes in detail the policy regarding data sharing, which is through a system of managed open access. Full instructions for applying for data access can be found here: http://www.bristol.ac.uk/alspac/researchers/access/. The ALSPAC study website contains details of all the data that are available (http://www.bristol.ac.uk/alspac/researchers/our-data/).
