# Supplementary material for: DXA-measured truncal adiposity in adolescence but not in childhood longitudinally predicts worsening cardiac outcomes
Source: Eur J Endocrinol. 2026 Apr 14;194(4):454–66. doi: 10.1093/ejendo/lvag044 (PMC13078852; doi:10.1093/ejendo/lvag044)
Supplement: lvag044_Supplementary_Data [file lvag044_supplementary_data.pdf]

Supplemental Appendix

DXA-measured Truncal Adiposity in Adolescence but not in Childhood Longitudinally Predicts Worsening Cardiac Outcomes

Andrew O. Agbaje MD, MPH, PhD[1,2]

[1]*Institute of Public Health and Clinical Nutrition, School of Medicine, Faculty of Health Sciences, University of Eastern Finland, Kuopio, Finland*

[2]*Children's Health and Exercise Research Centre, Department of Public Health and Sports Sciences, Faculty of Health and Life Sciences, University of Exeter, Exeter, UK.*

Address correspondence to:
Andrew O. Agbaje, MD, MPH, PhD, FACC, FESC, FAHA, FNYAM, Cert. Clinical Research (*Harvard*)
Professor *(associate)* of Clinical Epidemiology and Child Health
Principal Investigator urFIT-child research group. https://urfit-child.com/
Institute of Public Health and Clinical Nutrition, School of Medicine, Faculty of Health Sciences, University of Eastern Finland, Kuopio Campus.
Address: Yliopistonranta 8, P.O. Box 1627, 70211 Kuopio, Finland
E-mail: andrew.agbaje@uef.fi

Supplemental Table 1 Mean (SD) of body composition from ages 9 to 24 years.

Age	**9 years**		**11 years**		**15 years**		**17 years**		**24 years**	
N = 1803	*Mean*	*SD*	*Mean*	*SD*	*Mean*	*SD*	*Mean*	*SD*	*Mean*	*SD*
**Total body fat mass (kg)**	8.59	4.98	11.80	6.61	15.42	8.96	18.23	10.33	23.18	10.47
**Trunk fat mass (kg)**	3.46	2.43	5.02	3.31	7.2	4.77	9.34	5.71	11.19	6.0
**Lean mass (kg)**	24.41	3.12	29.67	4.29	42.46	8.33	45.62	9.78	47.65	9.70
**Body mass index (kg/m^2)**	17.67	2.81	19.06	3.32	21.40	3.47	22.79	3.96	24.87	4.85

Supplemental Table 2 Longitudinal associations of increased body composition from ages **9 – 24 years** with changes in left ventricular mass and diastolic function at age 24 years.

**N=1803**	**LVMI$^{2.7}$(g/m$^{2.7}$)**		**RWT (cm)**		**LVDF (E/A)**		**LVFP (E/e′)**	
Ages 9 – 24 years	***β** (95% CI)*	*p-value*	***β** (95% CI)*	*p-value*	***β** (95% CI)*	*p-value*	***β** (95% CI)*	*p-value*
**Total body fat mass (kg)**	-0.463 (-0.490 – -0.437)	**<0.001**	0.000 (-0.001 – 0.000)	**<0.001**	-0.004 (-0.005 – -0.003)	**<0.001**	0.000 (-0.002 – 0.003)	0.662
**Trunk fat mass (kg)**	-0.766 (-0.812 – -0.720)	**<0.001**	0.001 (-0.001 – 0.000)	**<0.001**	-0.008 (-0.010 – -0.006)	**<0.001**	-0.003 (-0.007 – 0.001)	0.153
**Lean mass (kg)**	0.325 (0.303 – 0.348)	**<0.001**	0.002 (0.002 – 0.002)	**<0.001**	-0.005 (-0.006 – -0.004)	**<0.001**	-0.007 (-0.008 – -0.005)	**<0.001**
**Body mass index (kg/m^2)**	0.238 (0.208 – 0.268)	**<0.001**	-0.001 (-0.001 – -0.001)	**<0.001**	0.002 (0.000 – 0.003)	**0.029**	0.009 (0.007 – 0.012)	**<0.001**

Model was adjusted for sex and other time-varying covariates measured at both baseline and follow-up such as age, high-sensitivity C-reactive protein, heart rate, systolic blood pressure, insulin, glucose, smoking status, family history of hypertension/diabetes/high cholesterol/vascular disease, socioeconomic status, sedentary time, light physical activity, moderate to vigorous physical activity, low-density lipoprotein cholesterol, high-density lipoprotein cholesterol, triglyceride and fat mass or lean mass, depending on the predictor. Regression coefficients ***(β)*** were computed from generalized linear mixed-effect model for repeated measures; CI, confidence interval; LVDF, left ventricular diastolic function; LVFP, left ventricular filling pressure; LVMI$^{2.7}$, left ventricular mass indexed for height$^{2.7}$; RWT, relative wall thickness, A 2-sided P-value <0.05 is considered statistically significant. Multiple testing was corrected with Sidak correction. Multiple imputations (20-cycles) were used to account for missing variables. A 1-unit change in exposure is associated with 1-unit change in the outcome.

Supplemental Table 3 Longitudinal associations of increased body composition from ages **17 – 24 years** with changes in left ventricular mass and diastolic function at age 24 years.

**N=1803**	**LVMI$^{2.7}$(g/m$^{2.7}$)**		**RWT (cm)**		**LVDF (E/A)**		**LVFP (E/e′)**	
Ages 17 – 24 years	***β** (95% CI)*	*p-value*	***β** (95% CI)*	*p-value*	***β** (95% CI)*	*p-value*	***β** (95% CI)*	*p-value*
**Total body fat mass (kg)**	0.046 (0.021 – 0.070)	**<0.001**	0.000 (0.0001 – 0.000)	**0.001**	-0.002 (-0.003 – -0.001)	**0.001**	0.000 (-0.002 – 0.002)	0.687
**Trunk fat mass (kg)**	0.094 (0.053 – 0.134)	**<0.001**	0.000 (0.000 – 0.001)	**<0.001**	-0.004 (-0.006 – -0.002)	**<0.001**	-0.003 (-0.007 – 0.001)	0.177
**Lean mass (kg)**	0.285 (0.267 – 0.303)	**<0.001**	0.000 (0.000 – 0.001)	**<0.001**	0.0001 (-0.001 – 0.001)	0.968	-0.002 (-0.004 – 0.000)	0.108

Model was adjusted for sex and other time-varying covariates measured at both baseline and follow-up such as age, high-sensitivity C-reactive protein, heart rate, systolic blood pressure, insulin, glucose, smoking status, family history of hypertension/diabetes/high cholesterol/vascular disease, socioeconomic status, sedentary time, light physical activity, moderate to vigorous physical activity, low-density lipoprotein cholesterol, high-density lipoprotein cholesterol, triglyceride and fat mass or lean mass, depending on the predictor. Regression coefficients ***(β)*** were computed from generalized linear mixed-effect model for repeated measures; CI, confidence interval; LVDF, left ventricular diastolic function; LVFP, left ventricular filling pressure; LVMI$^{2.7}$, left ventricular mass indexed for height$^{2.7}$; RWT, relative wall thickness, A 2-sided P-value <0.05 is considered statistically significant. Multiple testing was corrected with Sidak correction. Multiple imputations (20-cycles) were used to account for missing variables. A 1-unit change in exposure is associated with a higher or lower outcome unit.

Supplemental Table 4 Longitudinal associations of increased body composition from ages **9 – 17 years** with changes in left ventricular mass and diastolic function from ages **17 – 24 years**

**N=1803**	**$LVMI^{2.7}$($g/m^{2.7}$)**		**RWT (cm)**		**LVDF (E/A)**		**LVFP (E/e′)**	
Ages 9 – 17 years	***β** (95% CI)*	*p-value*	***β** (95% CI)*	*p-value*	***β** (95% CI)*	*p-value*	***β** (95% CI)*	*p-value*
**Total body fat mass (kg)**								
Model 1	0.591 (0.583 – 0.599)	**<0.001**	0.002 (0.002 – 0.002)	**<0.001**	-0.008 (-0.009– -0.008)	**<0.001**	0.014 (0.013 – 0.014)	**<0.001**
Model 2	-0.063 (-0.417 – 0.292)	0.728	0.001 (0.001 – 0.001)	**<0.001**	-0.313 (-0.333 – -0.293)	**<0.001**	-0.168 (-0.212 – -0.124)	**<0.001**
**Lean mass (kg)**								
Model 1	0.222 (0.217 – 0.227)	**<0.001**	0.000 (0.000 – 0.0001)	**<0.001**	-0.004 (-0.005 – -0.004)	**<0.001**	0.003 (0.003 – 0.004)	**<0.001**
Model 2	0.144 (0.138 – 0.149)	**<0.001**	0.022 (0.018 – 0.026)	**<0.001**	-0.093 (-0.123 – -0.063)	**<0.001**	-0.001 (-0.413 – -0.281)	**<0.001**

Model 1 was unadjusted. Model 2 was adjusted for sex and other time-varying covariates measured at both baseline and follow-up such as age, high-sensitivity C-reactive protein, heart rate, systolic blood pressure, insulin, glucose, smoking status, family history of hypertension/diabetes/high cholesterol/vascular disease, socioeconomic status, sedentary time, light physical activity, moderate to vigorous physical activity, low-density lipoprotein cholesterol, high-density lipoprotein cholesterol, triglyceride and fat mass or lean mass, depending on the predictor. Regression coefficients ***(β)*** were computed from generalized linear mixed-effect model for repeated measures; CI, confidence interval; LVDF, left ventricular diastolic function; LVFP, left ventricular filling pressure; $LVMI^{2.7}$, left ventricular mass indexed for $height^{2.7}$; RWT, relative wall thickness, A 2-sided P-value <0.05 is considered statistically significant and is bolded. Multiple testing was corrected with Sidak correction. Multiple imputations (20-cycles) were used to account for missing variables. A 1-unit increase in exposure is associated with the point estimate increase in the outcome.
